# The UK National Screening Committee’s position on child–parent screening for familial hypercholesterolaemia

**DOI:** 10.1177/09691413211025426

**Published:** 2021-07-22

**Authors:** David S Wald, Dermot Neely

**Affiliations:** DW is also Professor of Cardiology at Barts Heart Centre, St Bartholomew’s Hospital, London, UK, and Chair of the Familial Hypercholesterolaemia Expert Advisory Group, NHS England & Improvement. DN is a member of the Expert Advisory Group and National Adviser for Familial Hypercholesterolaemia, Academic Health Sciences Network North East and North Cumbria; 1 Population Health Research Institute, St George's University of London, London, UK; 2 Academic Health Sciences Network for the North East and North Cumbria, The Campus for Ageing and Vitality, Newcastle upon Tyne, UK

Familial hypercholesterolaemia (FH) is an inherited condition, affecting about 1 in 250 individuals, that results in high cholesterol and a high risk of fatal and non-fatal heart attacks at a young age.^1^ Only an estimated 7% of the 260,000 individuals with FH in the UK have been identified. Child–parent screening (CPS) is a method of identifying children and their parents with FH and supports cascade testing to identify other relatives with FH (child–parent cascade screening).^2^,^3^ Affected individuals have about a 100-fold excess risk of ischaemic heart disease between ages 20 and 39,^1^ which is largely reversed by starting a low saturated fat diet and preventive medication in childhood.^4^

In February 2020, the UK National Screening Committee (NSC) rejected the proposal for screening children for FH. In July 2020, National Health Service (NHS) England and NHS Improvement took a contrary view and chose to introduce CPS on a pilot basis as part of their Long-Term Plan to increase FH identification for the prevention of premature ischaemic heart disease.^5^

The NSC responded by setting up an Ethics Task Group. The ethical issues had already been considered by two National Research Ethics Committees prior to the Medical Research Council (MRC)-funded Child–parent Screening study in 2012. Neither committee found any ethical concern. It is not standard practice to reassess ethical issues unless new evidence arises that would alter the conclusions from previous ethical review. In the MRC-funded study, 10,095 children were screened at one to two years of age during routine immunisation visits at 92 English general practices. The method was found to be feasible, acceptable, safe and effective, with no new ethical issues raised.^2^ It is also cost effective.^6^ CPS has been independently assessed and introduced on a pilot basis in Western Australia and found to be similarly acceptable, feasible and cost effective.^7^,^8^

In spite of this, specific questions were asked by the NSC through its newly established Ethics Task Group on 26 January 2021 at a joint meeting of the NSC and NHS England and Improvement. The questions and the answers given are shown in Table 1. A key question that was not asked, but should have been, is whether it would be ethical not to introduce CPS given current knowledge and the large number of premature deaths from ischaemic heart disease that would be prevented. This omission is itself concerning.

**Table 1.** Questions on the child–parent screening (CPS) for familial hypercholesterolemia (FH) pilot programme posed by the UK National Screening Committee Ethics Task Force to NHS England and Improvement on 26 January 2021, and answers given.

UK National Screening Committee questions	NHS England and Improvement responses
Do you regard the pilot programme as research?	No, it is service implementation.
Is the primary aim of the pilot programme to confer health benefit to the screened children, or the adults, or both?	Both.
What is the justification for choosing to screen at age 1–2 years rather than closer to the age when statin treatment can be started?	There are at least 6 reasons:(i) It is the most accurate time in life to test.^8^(ii) Early dietary intervention benefits the whole family and particularly children whose tastes are developing and can be established for life. (iii) Early dietary intervention with a low saturated fat diet (at about 1 year) has been shown in a randomized trial to result in a healthier diet and lower LDL cholesterol levels at age 20, compared with no dietary intervention.^4^ (iv) The method of screening child and parent together has the benefits of simplicity (heel prick versus venous sample), effectiveness (80 new FH individuals identified per 10,000 children screened) and acceptability (84% uptake when linked to 1 year immunisation).^2^(v) One parent stands to benefit from immediate statin treatment; preventing a premature death of parent is a clear benefit to the child. (vi) Parental experience with preventive medication will be readily accepted by the child when he/she needs such treatment.
Could test accuracy studies in older children be undertaken in parallel to the pilot to contribute to discussion on the optimum age for screening?	Yes, but not needed because this is already known.^8^
Have you considered alternative strategies, and ages at which to screen children, to meet the Long-Term Plan targets?	Yes. None are as effective or available as CPS.
How will you mitigate any equity issues raised by the protocol? e.g. the potential for childless adults and parents who decline the immunisation appointment to be excluded.	CPS has been shown to be inclusive and widely adopted by families of different ethnic backgrounds. ^9^ Parents who decline immunisation can still be offered CPS and vice versa. Between 10 and 20% of adults are childless - similar to the proportion of breast cancers missed by screening at age 50. Almost all interventions miss some who stand to benefit. CPS identifies more FH than other available methods.
Have you considered the potential harms and benefits of a significant delay between diagnosis and the age at which treatment can be commenced in children?	Yes we have. There is no delay because the interval between identification and drug treatment is part of the protocol and expected. During this interval, dietary intervention begins early and this has been shown to benefit children. The whole family benefits from the incentivised advice to eat a healthy diet. Early identification of a screen-positive child allows preventive treatment to start in the parent immediately; delayed diagnosis will delay prevention and miss avoidable parental deaths. Preventing the premature death of a parent benefits the child and society; the interests of child and parent concur.
Are you collecting data on cost effectiveness and will you be comparing this to other strategies or ages at which screening might be offered?	No. Cost-effectiveness analyses have already been undertaken and CPS is more cost effective than other strategies.^10^
Have you consulted with people working in the delivery of immunisations about how this would work in practice?	Yes.
Would you capture any effects on the uptake of immunisation?	No. This was assessed in the MRC National Demonstration Project and immunisations went up, not down, during the screening years. ^2^ There are no plans to repeat this assessment.
What is the process for obtaining consent (providing information, time to consider)? What are you asking parents to consent to? Is it possible to see the consent form?	Verbal consent to test their child and later to test the parent. Information will be sent before immunisation (a leaflet and a multilanguage video (www.explainmyprocedure.com/cps. ) and verbal consent recorded by a nurse.
Will the time taken for seeking consent, as well as the time taken to do the sample, be included in the evaluation?	Yes.
Is there provision for training of the individuals taking the sample?	Yes.
When, how and by whom will parents be told the result, and will parents be told an actual value if requested?	In writing by practice staff after the screening visit, and yes.
What information about cholesterol will be given to concerned parents of children below the threshold for mutation testing, and are cholesterol reference ranges available for this age group to guide advice?	They will be told the result is negative and therefore not at high risk of ischaemic heart disease, and yes, from the MRC study.
Will you be using the same testing kit as in the previous study of 10,000?	Either the same or better.
Have you considered your obligations in relation to reporting findings and offering testing to other family members? For example, where parents are resistant to contacting other family members, or if other family members are not easily contacted (e.g. if child is adopted or family members reside in other countries).	Yes, tracing family members once a proband is found is part of usual care. ^11^
You will be taking and storing a blood sample in case cholesterol is above the threshold.	Incorrect – no samples are stored.
If children are referred to specialist FH clinics, has the additional resource to advise and provide support to the children and families been considered, e.g. around dietary measures? If GPs are expected to do this, has there been consultation with them or representative bodies?	Usual care involves advice from a dietician or suitably trained person, which will be needed in no more than one extra family per general practice per year. If there are any circumstances where a GP needs specialist support, referrals can be made through usual care pathways.

LDL: low-density lipoprotein; MRC: Medical Research Council; GP: general practitioner.

The NSC seems to take the view that the child does not benefit from screening; only the parent benefits and the child is simply a conduit to the parent. However, both child and parent benefit. The child begins a healthy low saturated fat diet from age 1 year. Randomized trial evidence has shown that dietary advice started at eight months of age results in a healthier diet at age 20 years and lower low-density lipoprotein cholesterol (LDL-C) levels than children randomized to no dietary advice.^9^ The trial results support the expected – that it is easier to introduce good dietary habits early, when tastes are developing, than break bad ones later. But the study goes further and shows that the benefits are sustained. Since LDL-C is the underlying cause of ischaemic heart disease, a lower LDL-C level reduces risk. A healthy diet that benefits a child also benefits the parent, and the wider family, because eating tends to be a shared activity. Early identification of the child therefore benefits the whole family, not just the child, and there are no ethical issues raised by family-based prevention.

For most children found to have FH at age 1 year, there will be about nine years between identification and the start of statin treatment. The NSC sees this as an ethical problem but it is not. There is no “delay” because the interval is expected as part of the screening protocol. The identified parent starts drug treatment immediately and preventing the premature death of a parent is an important benefit to the child. Both child and parent benefit, and so too does society. Delaying screening until age 10 would be unethical if there were a practical earlier available opportunity to screen, because delay will result in avoidable parental illness and death.

The NSC also overlooks the benefit of CPS on the identification of children with homozygous FH, where an abnormal cholesterol-raising gene is inherited from both parents. This has a prevalence, about 1 in 250,000 comparable to other serious rare inherited disorders routinely screened for at birth.^10^ Homozygous FH causes extremely high LDL-C levels and death or disability from ischaemic heart disease in most cases by age 20 years. Pharmacological treatment needs to start by one year of age and, together with apheresis started at about age 5, is effective in reducing this risk.^10^ CPS is the only available systematic method for identifying such children. It would be arguably unethical, and certainly inequitable, to deny society this preventive opportunity when it is offered for other comparable rare conditions.

For the 2500 children with heterozygous FH born each year in the UK, evidence supports early initiation of statin treatment. A 20-year follow-up study of children who were previously participants in a placebo-controlled trial evaluating the two-year efficacy and safety of pravastatin showed that those who remained on treatment until age 40 had LDL-C levels comparable to their unaffected siblings and avoided ischaemic heart disease events completely,^4^ effectively abolishing the high excess risk that applies to untreated individuals with FH at this age.^1^ This contrasted with their FH-positive parents who started statins later in life and in whom fatal or non-fatal ischaemic heart disease events were observed before age 40 in about 25% of cases.^4^ Mendelian randomisation studies confirm that prolonged exposure to lower LDL-C beginning early in life is associated with a substantially greater reduction in the risk of ischaemic heart disease than the practice of lowering LDL-C beginning later in life.^11^ The available evidence is consistent and clear; that early identification of a child provides an opportunity to prevent serious disease in both child and parent together.

 CPS, as with all screening programmes, causes anxiety, but providing the anxiety is limited to those people who will benefit from accepting the offer of an effective remedy it has a positive and useful effect. In CPS, a child is FH-positive if their blood cholesterol is high (≥95th centile) and an FH mutation is identified or very high (≥99th centile) on two measurements several months apart. This method, determined from paired analysis of cholesterol and FH mutations in over 10,000 children, recognises that in itself a deleterious change in the sequence of an FH-related gene is insufficient to identify a group in the population at high risk of inherited premature ischaemic heart disease that can successfully and safely be prevented by reducing LDL-C with diet and statins. While no screening test is perfect, the CPS approach minimises false positives thereby limiting initial anxiety to those who stand to gain by avoiding the excess risk of a premature heart attack. A UK study showed that more than 90% of parents who had one child screened by CPS said they would screen a second child if screening was offered.^12^ Similar public support for CPS has been demonstrated in Australia.^7^

We believe the NSC was mistaken in rejecting child screening for FH on scientific grounds.^13^ The decision to commission an Ethics Task Group to re-examine the case for CPS is a strange one, and the group’s membership is surprising because the group is not independent of the NSC, which is of concern given that the NSC had already taken a negative position on CPS. This gives some grounds for suspicion that the NSC set up the group in an attempt to reinforce their decision to reject CPS; invoking unethical practice is sometimes the position taken when the scientific argument has been lost. Whatever the motivation and intention of setting up the Ethics Task Group, it gives an impression of undue defensiveness, and is not without the risk of causing harm; even raising the possibility of an ethical issue has harmful consequences by creating hesitancy among clinical and laboratory staff who are tasked with implementing NHS England’s Long-Term Plan.

Rather than pondering ethical issues which have been resolved already, the NSC would be well advised to reconsider their decision. Given the known benefits of CPS set against no material evidence of harm, to block or further delay its introduction would constitute a lost opportunity in the prevention of premature ischaemic heart disease due to FH.
